# Supplementary material for: Action potential metrics and automated data analysis pipeline for cardiotoxicity testing using optically mapped hiPSC-derived 3D cardiac microtissues
Source: PLoS One. 2023 Feb 6;18(2):e0280406. doi: 10.1371/journal.pone.0280406 (PMC9901774; doi:10.1371/journal.pone.0280406)
Supplement: S1 Text — This supplemental document includes S1 to S5 Figs and Python routines of segmentation, baseline subtraction, bilinear filtering, rise time and repolarization measurements using moving average subtraction. (DOCX) [file pone.0280406.s001.docx]

**Supplemental Material**

**Action potential metrics and automated data analysis pipeline for cardiotoxicity testing using optically mapped hiPSC-derived cardiac microtissues.**

Arvin Soepriatna^2,*^, Allison Navarrete-Welton^1,*^, Tae Yun Kim^1^, Mark C Daley, Peter Bronk^1^, Celinda M. Kofron^1^, Ulrike Mende^1^, Kareen L.K. Coulombe^2^, Bum-Rak Choi^1^

# AP metric changes under I_to_ and I_Ks_ block: Computer simulation study

The impact of I_to_ block on AP shape is complex. Although I_to_ is a repolarizing current, the block of I_to_ with the default parameter setting of O’Hara human AP model^1^ caused increased AP upstroke amplitude but shortened APD (panel A). The APD shortening under I_to_­ block was due to ‘knock-on’ effect of I_to­_ on other currents such as I_Ca_ ^2^. Overall, AP metric changes under I_to_­ (panel B) and I_Ks_ block (panel C) were relatively small compared to other major ionic currents such as I_Na_, I_Ca_, or I_Kr_ block. Even complete block of I_to_ and I_Ks_ in human AP model showed less than 10% change (panel B and C). This simulation indicates that the detection of I_to_ and I_Ks_ block may require optimized protocol such as fast pacing rate or beta-adrenergic stimulation to increase the effect of I_Ks_ on APD.


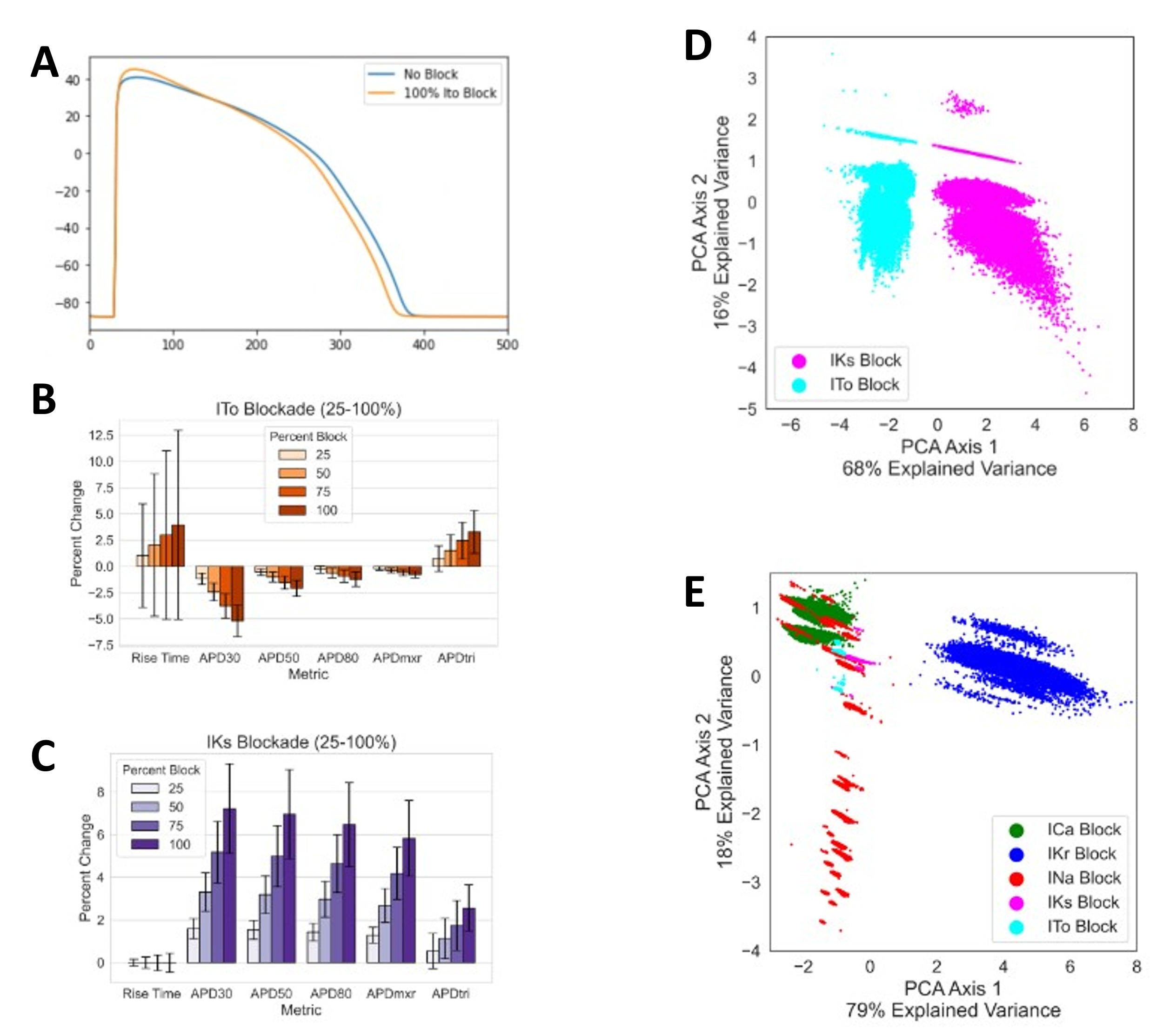


**Supplemental Figure S1. AP metric changes under I_to_ and I_Ks_ block: computer simulation.** (A) Sample traces of AP under normal (blue) and I_to_ block (red). (B) AP metric changes after I_to_ block. Very small changes were seen from I_to_ block in O’hara human AP model. (C) I_Ks_ block. I_Ks_ block caused relatively same changes in all APD metrics including APD_30_, APD_50­,_ APD_80_ and APD_MXR_. (D/E) PCA plot created using I_to_ and I_Ks_ simulation data only (D) and together with I_Na_, I_Ca_, I_Kr_ blocks (E). I_to_ and I_Ks_ block can be separated along the PCA axis 1 in direction of APD prolongation.

**Supplemental Table 1. PAC axes and explained variance ratio of the computer modeling data in Supplemental Figure S1 including I_to_ and I­_Ks_ block**

| PCA Axis | APD_30_ | APD_50_ | APD_80_ | APD_mxr_ | APD_tri_ | Rise Time | Delay | Explained Variance Ratio |
| --- | --- | --- | --- | --- | --- | --- | --- | --- |
| 1 | 0.49 | 0.49 | 0.49 | 0.49 | -0.11 | -0.16 | N/A | 0.68 |
| 2 | 0 | -0.03 | -0.04 | -0.15 | -0.99 | 0 | N/A | 0.16 |
| 3 | 0.06 | 0.08 | 0.09 | 0.09 | 0 | 0.99 | N/A | 0.15 |

# Maps of APD and AP upstroke rise time

Typically, we imaged 5x5 mm^2^ field of view that covers 4x4 microtissues. APD_MXR_ and rise time were calculated from each pixels using the same algorithm described in Results section. The mean standard deviation of APD within the same microtissue (panel A) is 1.6 ms while standard deviation of APDs between microtissues is 10.6 ms (n=34), indicating that APDs in the same microtissue are similar and the mean value of APD per microtissue can be used to evaluate drug effects.

The mold of 5x7 microtissues was paced with two linear platinum electrodes. This field stimulation caused hyperpolarization near anode and depolarization near cathode (panel B traces), which is seen in heart tissue, known as virtual electrode effect ^3^. Our microtissues have 3D spheroid shapes that are sufficiently large to have restricted extracellular space within the microtissues, causing both hyperpolarization and depolarization within the same microtissue. This produced a heterogeneous rise of action potential upstroke, with a faster rise in the hyperpolarized region than the depolarized region (panel B). As a result, minor changes in AP upstroke can be masked by the virtual electrode effect and only large changes from Na^+^ channel blockers can be detected.


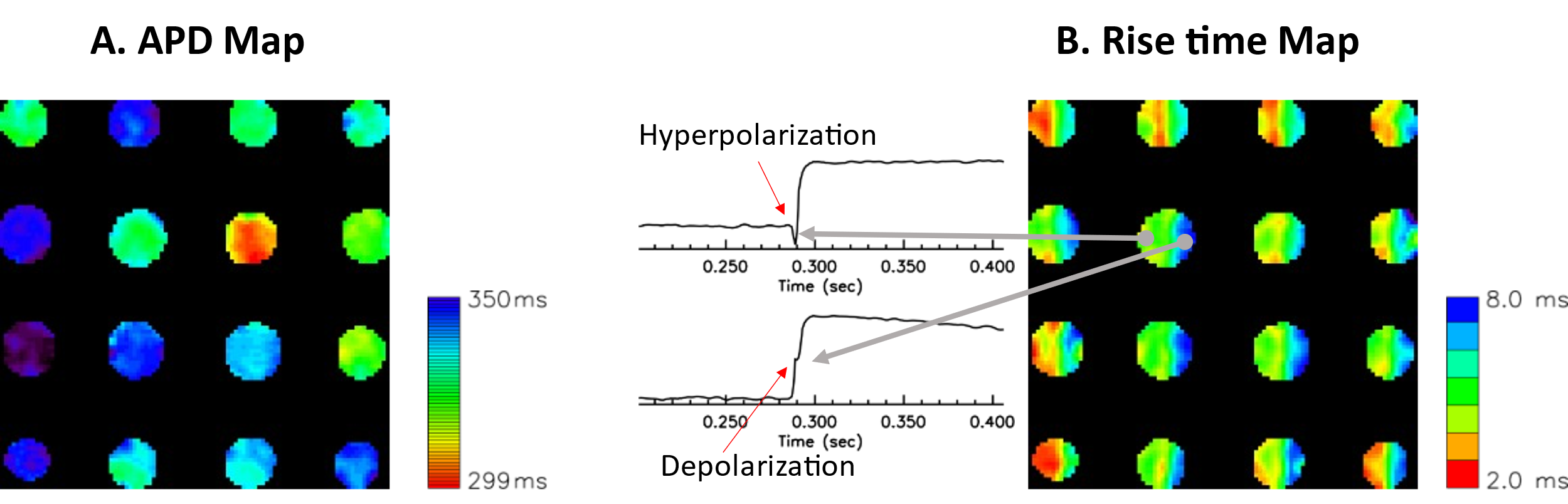


**Supplemental Figure S2. Maps of APD and AP upstroke rise time.** (A) Map of APD. APDs are relatively uniform within the same microtissue. The mean standard deviation of APD within the same microtissue is 1.6 ms while standard deviation of APDs between microtissues is 10.6 ms (n=34). (B) Sample traces and map of AP upstroke rise time. The field stimulation caused a voltage gradient (upper trace: hyperpolarization, lower trace: depolarization), which affects the rise time of AP.

# Parameter selection for bilateral filtering

The bilateral filtering ^4^ is determined by two parameters, σ_g_ for conventional gaussian filter and σ_I_ for filtering based on the intensity differences as follows.

$$I(t)=\frac{1}{W_{p}}\sum G_{\sigma s}(\left\| p-q \right\|)G_{\sigma r}(I_{p}-I_{q})I_{q}$$

$$W_{p}=\sum G_{\sigma s}(\left\| p-q \right\|)G_{\sigma r}(I_{p}-I_{q})$$

Bilateral filtering adds additional term to adjust the weights of points depending on the similarity of intensity (G_sr_(I_p_-I_q_)). Increasing σ_g_ blurs the AP upstrokes significantly, even with small σ_g_ size (>4, cyan, orange and red color traces), which causes the rise time measurements to be significantly longer than the true AP upstroke (panel B). This blurring of the AP upstroke can be avoided by incorporating the additional parameter σ_I_ in Bilateral filtering. If the intensity difference between the reference time point and the smoothing time point is small, the two values are averaged based on Gaussian filtering. However, if the two values are greater than σ_I,_ the smoothing effect is reduced in Bilateral filtering. If σ_I_ is set to the amplitude of the AP upstroke, Bilateral filtering is equivalent to Gaussian filtering (panel C-D). The optimal σ_I_ can be set to the basal noise level (0.15 in this example), which is sufficient to reduce noise while preserving the sharpness of AP upstroke (panel E).


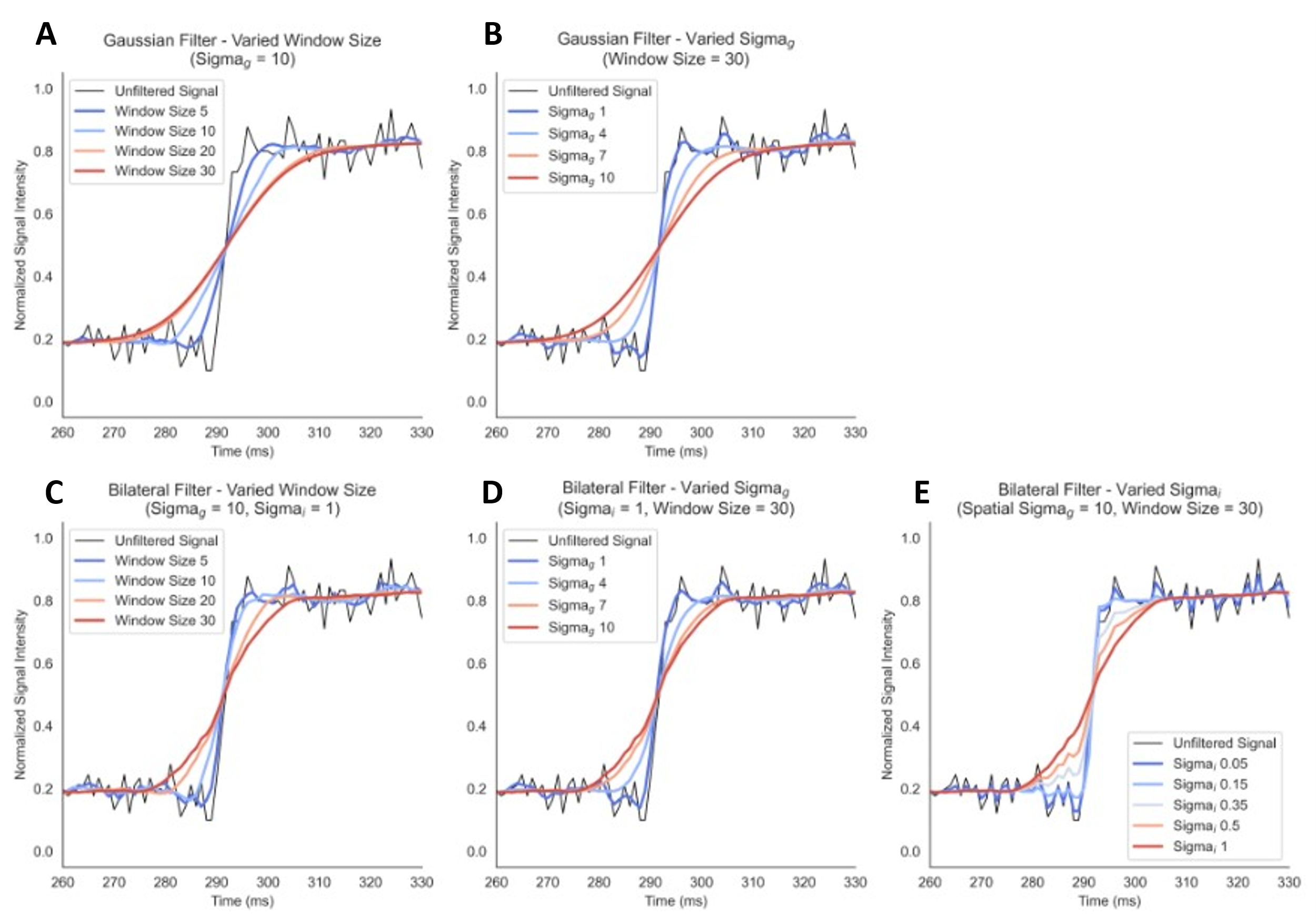


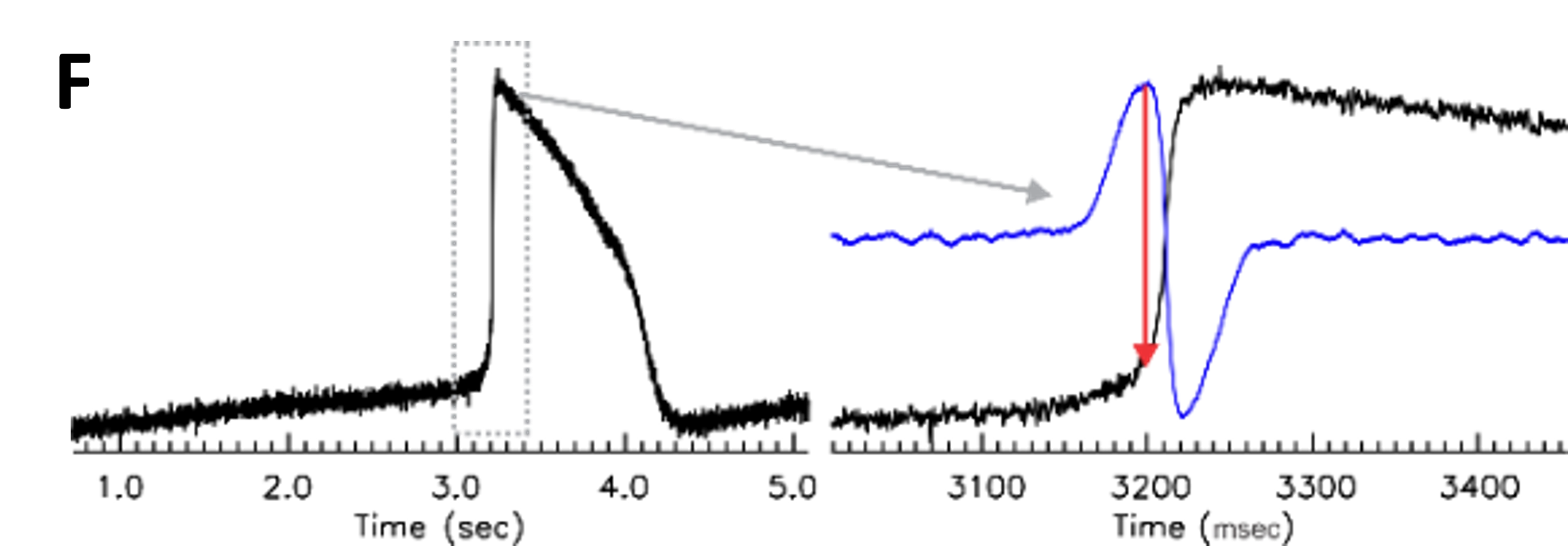


**Supplemental Figure S3. Impact of bilateral filter parameters on blurring and edge preservation.** (A-B) Gaussian filtering. σ_g_ (right) was varied from 1 to 10 to examine its influence on blurring and edge preservation. (C-E) Bilateral filtering. By adjusting σ_I_, the blurring on the AP upstrokes can be avoided. The optimal σ_I_ can be set to the basal noise level (σ_i_ = 0.15, cyan color trace in panel E). (F) Increased automaticity and detection of AP takeoff using bilateral and moving average subtraction. Spontaneous beating rate of 3D microtissues is very slow and cannot be detected easily during 8 sec recording time. The automaticity in this example was induced by quinidine (100 μM). A slow rise of pacemaker potential is shown before a full AP is triggered. Bilateral filtering and moving average subtraction can successfully detect the proper takeoff time point.

# Experimental data of I_Ks_ blocker (chromanol 293B) on AP metrics


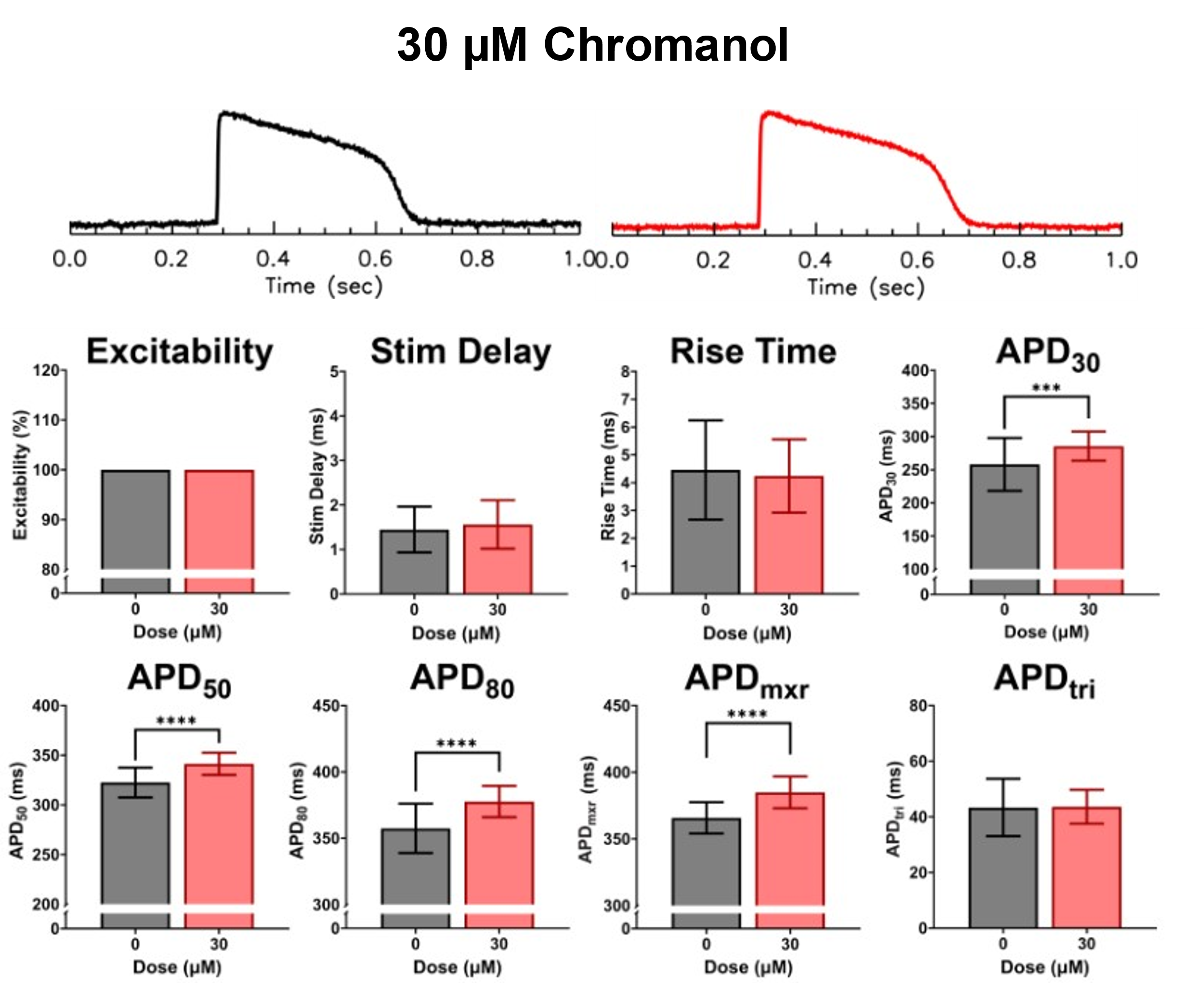


**Supplemental Figure 4. AP metric changes by I_Ks_ blocker**. Chromanol blocks I_Ks_ (IC50 = 2 ~ 6 µM) and also I_to_ (IC50 = 24 µM). We tested 30 µM concentration, that blocks I_Ks_ completely to have large effect on APD. At this concentration, chromanol significantly prolong APD_50_ APD_80_, and APD_MXR_ without altering APD_tri_ as predicted by the computer simulation shown in panel C of Supplemental Figure S1.

# Comparison of PCA plots from simulation and experimental data

Our analysis indicates that two PCA axes can be used to visualize AP metric changes under I_Na_, I_Ca_, and I_Kr_ blocks. Supplemental Figure S4 shows superimposed PCA plots of both simulation and experimental data. PCA plots show that both experimental (darker colors) and computer modeling (lighter colors) show similar cluster patterns. Notably, the Flecainide cluster falls midway between TTX and E4031 clusters reflecting that its effect combines an I_Na_ block similar to TTX and an I_Kr_ block similar to E4031.


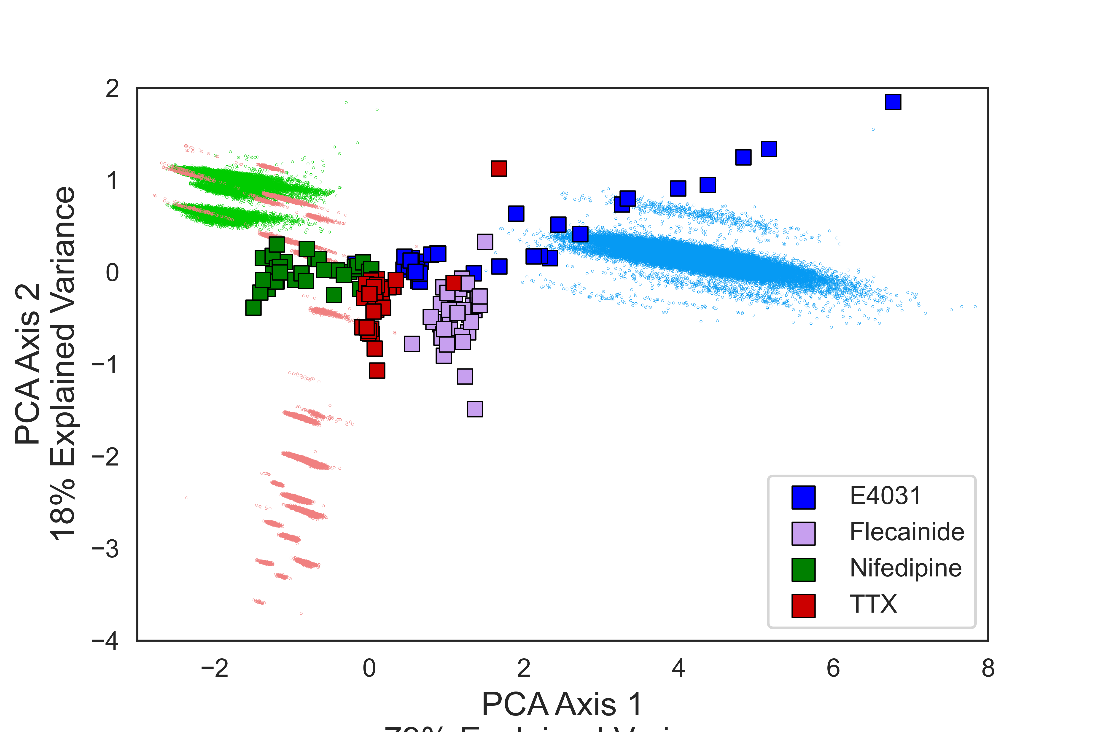


**Supplemental Figure S5. Superimposed PCA plots of computer simulation and experimental data of ion channel block.** Computer simulation (dots) and experimental data (squares) plotted on the PCA axes produced with the simulation data (see Table 2 in main text).

# Python scripts for signal processing and automated data analysis

Segmentation using minimum cross entropy algorithm

We used the cross entropy implantation available in python scikit-image package. This package is available from <https://scikit-image.org>. The images of either fluorescence intensity or FFT_max_ were smoothed with 3x3 window size and normalized from min to max into 256 levels. The Skimage package has two routines available for minimum cross-entropy thresholding, iterative algorithm (**threshold_li**) and calculation of cross entropy from defined threshold (**_cross_entropy**). Our image consists of 100x100 pixels in 256 gray scale level, which is not computationally intensive. Therefore, we used **_cross_entropy** routine to calculate at every gray scale level from 1 to 254) and tried to find the threshold when the cross entropy value is minimum as follows.

**import** **numpy** **as** **np**

**import** **matplotlib.pyplot** **as** **plt**

**from** **skimage** **import** data

**from** **skimage** **import** filters

**from** **skimage.filters.thresholding** **import** _cross_entropy

[thresholds](https://numpy.org/doc/stable/reference/generated/numpy.ndarray.html#numpy.ndarray) = [np.arange](https://numpy.org/doc/stable/reference/generated/numpy.arange.html#numpy.arange)(np.min([image](https://numpy.org/doc/stable/reference/generated/numpy.ndarray.html#numpy.ndarray)) + 1.5, np.max([image](https://numpy.org/doc/stable/reference/generated/numpy.ndarray.html#numpy.ndarray)) - 1.5)

[entropies](https://docs.python.org/3.9/library/stdtypes.html#list) = [_cross_entropy([image](https://numpy.org/doc/stable/reference/generated/numpy.ndarray.html#numpy.ndarray), t) **for** t in [thresholds](https://numpy.org/doc/stable/reference/generated/numpy.ndarray.html#numpy.ndarray)]

[optimal_camera_threshold](https://numpy.org/doc/stable/reference/arrays.scalars.html#numpy.float64) = [thresholds](https://numpy.org/doc/stable/reference/generated/numpy.ndarray.html#numpy.ndarray)[[np.argmin](https://numpy.org/doc/stable/reference/generated/numpy.argmin.html#numpy.argmin)([entropies](https://docs.python.org/3.9/library/stdtypes.html#list))]

Baseline Subtraction:

# Baseline correction using asymmetric least square fitting

# copyright © 2022, Bum-Rak Choi, Taeyun Kim, Allison Navarrete-Welton

# This program is free software: you can redistribute it and/or modify

# it under the terms of the GNU General Public License as published by

# the Free Software Foundation, either version 3 of the License, or

# (at your option) any later version.

#

# This program is distributed in the hope that it will be useful,

# but WITHOUT ANY WARRANTY; without even the implied warranty of

# MERCHANTABILITY or FITNESS FOR A PARTICULAR PURPOSE. See the

# GNU General Public License for more details.

#

# You should have received a copy of the GNU General Public License

# along with this program. If not, see <https://www.gnu.org/licenses/>.

#

def smooth(data, width = 40):

''' Smoothes signal using moving average function

Parameters

----------

data: input signal (1D numpy array of float32)

width: window size for moving average smoothing (int)

Returns

----------

smooth: signal smoothed via moving average method (1D numpy array of float32)'''

#average along sequential windows

half_width = int(width / 2)

new_data = [np.sum(data[i - half_width:i + half_width + 1]) for i in np.arange(half_width, len(data)-width)]

new_data = np.array(new_data) / (width - 1)

#concatenate with original data at array edges

smooth = np.concatenate([data[:half_width], new_data, data[len(data)-width:]])

return smooth

def make_second_diff_matrix(n):

''' Makes second difference matrix

Parameters

-----------

n: matrix dimension (int)

Returns

--------

d: second difference matrix (2D numpy array) '''

dn = np.ones(n)

dn[0] = 0

dn1 = np.ones(n-1)*-2

dn1[0] = -1

dn2 = np.ones(n-2)

d_combine = np.concatenate([dn, dn1, dn2])

d_index_x = np.concatenate([np.arange(n), np.arange(n-1), np.arange(n-2)])

d_index_y = np.concatenate([np.arange(n), np.arange(n-1)+1, np.arange(n-2)+2])

d = csr_matrix((d_combine, (d_index_x, d_index_y))).toarray()

return d

def make_asl_matrix(lamda, w):

'''Makes matrix terms that are part of the solution to the

minimization of the asymmetric least squares equation.

Parameters

-----------

lamda: factor that balances smoothness and data fit (float32)

w: weight vector (1D numpy array of float32)

Returns

--------

A: inverse of lamda_d_gram + weight matrix (2D numpy array of float32)

weights: diagonal matrix of weights (2D numpy array of float32)'''

n = np.size(w)

i = np.arange(n)

weights = csr_matrix((w, (i,i)), shape = (n,n)).toarray()

d = make_second_diff_matrix(n)

d_gram = np.matmul(d, d.transpose()) #Gram matrix of 2nd difference matrix

lamda_d_gram = d_gram*lamda

A = np.linalg.inv(weights + lamda_d_gram)

return A, weights

def baseline_adjust(data, lamda = 1000, p = 0.001, i_iter = 5, n_max = 124):

''' Finds baseline and corrects signal, normalizing as dF/F0. Method

inspired by Eleirs & Boelens, 2005. See Baek et al, Analyst 2015, 140, 251-252.

Parameters

-----------

data: signal (1D numpy array of float32)

lamda: weight parameter that balances the data fit with the smoothness in

the regularized least squares function (float32)

p: asymmetry parameter, recommended to set between 0.001-0.01 (float32)

i_iter: number of iterations to minimize cost function (int)

n_max: maximum number of points to downsample signal (int)

Returns

-----------

baseline_subtract: baseline-adjusted signal (1D numpy array of float32)'''

#resample to constrain signal length to n_max

n_res = np.size(data)

y = data

if n_res > n_max:

n = n_max

y = y[::int(n_res/n)][:n]

i_comp_flag = 1

else:

y = data

n = n_res

i_comp_flag = 0

y = smooth(y, 10)

w = np.ones(n)

A, W_mat = make_asl_matrix(lamda, w) #A = W + lamda_d_gram

baseline = np.matmul(np.matmul(A, W_mat), y)

#iterate to update weights and remove peaks from signal for fitting

for i in range(i_iter):

#weights are set near-zero in peak regions

w = p*(y > baseline) + (1-p)*(y <= baseline)

#update A with new weights before re-solving

A, W_mat = make_asl_matrix(lamda, w)

baseline = np.matmul(np.matmul(A, W_mat), y)

#interpolate to resize baseline-smoothed signal to original signal length

if i_comp_flag == 1:

baseline = zoom(baseline, n_res/len(baseline))

#correct for baseline by dividing dF/F0

baseline_subtract = -(data - baseline)/baseline

return baseline_subtract

Non-linear bilateral filtering

# Adaptation of Bilateral filtering to hiPSC-CM AP recordings

# copyright © 2022, Bum-Rak Choi, Taeyun Kim, Allison Navarrete-Welton

# This program is free software: you can redistribute it and/or modify

# it under the terms of the GNU General Public License as published by

# the Free Software Foundation, either version 3 of the License, or

# (at your option) any later version.

#

# This program is distributed in the hope that it will be useful,

# but WITHOUT ANY WARRANTY; without even the implied warranty of

# MERCHANTABILITY or FITNESS FOR A PARTICULAR PURPOSE. See the

# GNU General Public License for more details.

#

# You should have received a copy of the GNU General Public License

# along with this program. If not, see <https://www.gnu.org/licenses/>.

#

def bilateral_filter(data, window_size = 21, sigma_g = 10.0, sigma_i = 0.1):

'''Implements bilateral filtering, a method to preserve the sharpness

of AP upstrokes. Each pixel value is replaced by a normalized weighted

average of surrounding pixel values with the largest weights assigned

to nearby pixels with similar intensities.

Parameters

----------

data: input signal (1D numpy array of float32)

window_size: window size for gaussian filtering; default = 21, should be odd (int)

sigma_g: filtering parameter for gaussian filter; default = 10.0 (float32)

sigma_i: filtering parameter to adjust based on intensity differences;

default = 0.1 (10% level for a normalized trace case) (float32)

Returns

----------

filtered: filtered data (1D numpy array of float32)'''

#Set parameters

g_sd = -0.5/(sigma_g**2)

i_sd = -0.5/(sigma_i**2)

n = np.size(data)

#Make Gaussian filter

window_inds = np.arange(window_size) - (window_size - 1)/2

filter_arr = np.exp(window_inds**2 * g_sd)

g_filter = filter_arr / np.sum(filter_arr)

#Combine Gaussian & intensity filtration

temp = np.zeros(n)

normalize_factors = np.zeros(n)

for dist_idx in range(window_size - 1):

i_input = np.roll(data, dist_idx - int(window_size/2))

i_filter = np.exp((data - i_input)**2 * i_sd)

temp += g_filter[dist_idx] * i_filter * i_input

normalize_factors += i_filter * g_filter[dist_idx]

filtered = temp/normalize_factors #normalize

return filtered

Analysis Code (rise time, APDmxr, APD30, APD50, APD80, APDtri):

# Automated analysis routines to measure AP metrics

# copyright © 2022, Bum-Rak Choi, Taeyun Kim, Allison Navarrete-Welton

# This program is free software: you can redistribute it and/or modify

# it under the terms of the GNU General Public License as published by

# the Free Software Foundation, either version 3 of the License, or

# (at your option) any later version.

#

# This program is distributed in the hope that it will be useful,

# but WITHOUT ANY WARRANTY; without even the implied warranty of

# MERCHANTABILITY or FITNESS FOR A PARTICULAR PURPOSE. See the

# GNU General Public License for more details.

#

# You should have received a copy of the GNU General Public License

# along with this program. If not, see <https://www.gnu.org/licenses/>.

#

def smooth(data, width = 40):

''' Smoothes signal using moving average function

Parameters

----------

data: input signal (1D numpy array of float32)

width: window size for moving average smoothing (int)

Returns

----------

smooth: signal smoothed via moving average method (1D numpy array of float32)''’

#average along sequential windows

half_width = int(width / 2)

new_data = [np.sum(data[i - half_width:i + half_width + 1]) for i in np.arange(half_width, len(data)-width)]

new_data = np.array(new_data) / (width - 1)

#concatenate with original data at array edges

smooth = np.concatenate([data[:half_width], new_data, data[len(data)-width:]])

return smooth

def get_first(mask, start, end):

''' Find index of first 1 in a 1D array of 0s and 1s

Parameters

----------

mask: array of 0s and 1s (1D numpy array)

start: start index of search window (int)

end: end index of search window (int)

Returns

--------

int: index of first 1 in array (-1 if array is all zeros)

'''

non_zeros = mask[start:end].nonzero()

if np.any(non_zeros):

return int(non_zeros[0][0] + start)

else:

return -1

def get_peak_windows(data, default_n_peaks, z_criteria = 0.45):

''' Finds number of APs & windows around each AP peak using

signal first derivative

Parameters

----------

data: input signal (1D numpy array of float32)

default_n_peaks: estimated number of APs (int)

z_criteria: z-score threshold for peak identification (float)

Returns

----------

peak_windows: start and end indices for windows around each AP peak

(2D numpy array of ints)

n_peaks: number of APs (int) '''

deriv1 = np.diff(data)

arr_size = len(data)

peak_windows = np.zeros((2, default_n_peaks)) #allocate space

n_peaks = 0

#remove first and last regions (intensity may not be stable)

start_ind, end_ind = 10, arr_size - 10

#get Z-scores

std = np.std(deriv1[start_ind:end_ind])

z_scores = deriv1/std

#Calculate upper and lower z-score thresholds

gt_crit = z_scores > z_criteria

lt_crit = z_scores < -z_criteria

#Set windows around AP peaks

while start_ind < arr_size and n_peaks < default_n_peaks:

#Find next index where signal is greater than upper criteria

#This will be the start of the action potential peak window

first = get_first(gt_crit, start_ind, end_ind)

if first == -1:

return peak_windows.astype(int)[:, :n_peaks], n_peaks

if first > start_ind:

start_ind = first

n_peaks += 1

peak_windows[0, n_peaks - 1] = start_ind

#Find next index where signal is less than the lower criteria

#This will be the end of the action potential peak window

first = get_first(lt_crit, start_ind, end_ind)

if first == -1:

peak_windows[1, n_peaks - 1] = arr_size - 1

return peak_windows.astype(int)[:, :n_peaks], n_peaks

#Set end index

if first == start_ind:

start_ind = start_ind + 1

else:

start_ind = first

peak_windows[1,n_peaks - 1] = start_ind

return peak_windows.astype(int)[:, :n_peaks], n_peaks

def estimate_peak_idxs(data, peak_windows, n_peaks):

'''Uses maximum of first derivative signal to set approx peak indices

within previously estimated windows around each peak

Parameters

----------

data: input signal (1D numpy array of float32)

peak_windows: start and end indices for windows around each AP peak

(2D numpy array of ints)

n_peaks: number of APs (int)

Returns

----------

AP_peak_deriv1_inds: approximate indices of AP peaks (1D numpy array of ints)'''

AP_peak_deriv1_inds = np.zeros(n_peaks) #allocate space

if n_peaks > 0:

smoothed_deriv = np.diff(smooth(data, width = 100)) #Moving average smoothing

AP_peak_deriv1_inds = np.zeros(n_peaks) #Allocate space

#Set peak estimate at the maximum of the smoothed derivative in each peak window

for i in range(n_peaks):

ind_max = np.argmax(smoothed_deriv[peak_windows[0,i]:peak_windows[1,i]]).astype(int)

AP_peak_deriv1_inds[i] = peak_windows[0,i] + ind_max

return AP_peak_deriv1_inds.astype(int)

def find_AP_peaks(data, n_peaks, AP_peak_deriv1_inds, peak_window_size = 80):

'''Sets AP peak as maximum value within each search window

Parameters

----------

data: input signal (1D numpy array of float32)

n_peaks: number of APs (int)

AP_peak_deriv1_inds: indices of approximate AP peak locations (1D numpy array of ints)

peak_window_size: size of search window; default = 80 (int)

Returns

----------

AP_peak_inds: AP peak indices (1D numpy array of ints)

AP_peak_vals: AP peak values (1D numpy array of float32)'''

AP_peak_inds = np.zeros(n_peaks) #allocate space

AP_peak_vals = np.zeros(n_peaks)

n_frames = len(data)

for i in range(n_peaks):

#Set search window starting from the indices found using the

#first derivative criteria

start_ind = AP_peak_deriv1_inds[i]

end_ind = start_ind + peak_window_size

if start_ind < 0:

start_ind = 0

if end_ind >= n_frames:

end_ind = n_frames - 1

#Find maximum in search window

ind_max = np.argmax(data[start_ind:end_ind])

AP_peak_inds[i] = ind_max + start_ind

AP_peak_inds = AP_peak_inds.astype(int)

AP_peak_vals = data[AP_peak_inds]

return AP_peak_inds.astype(int), AP_peak_vals

def find_AP_takeoffs(data, n_peaks, AP_peak_deriv1_inds, takeoff_window = 40,

takeoff_smooth = 100):

'''Finds action potential takeoff points

Parameters

----------

data: input signal (1D numpy array of float32)

n_peaks: number of APs (int)

AP_peak_deriv1_inds: indices of approximate AP peak locations (1D numpy array of ints)

takeoff_window: window size before AP peak for takeoff; default = 40 (int)

takeoff_smooth: width of moving average smoothing function; default = 100 (int)

Returns

----------

AP_takeoff_inds: 1D numpy array of AP peak indices (ints)

AP_takeoff_vals: 1D numpy array of AP peak values (floats)'''

AP_takeoff_inds = np.zeros(n_peaks) #allocate space

AP_takeoff_vals = np.zeros(n_peaks)

MAS = smooth(data, takeoff_smooth) - data

for i in range(n_peaks):

#set search window before action potential peak chosen by 1st derivative

ref = AP_peak_deriv1_inds[i]

start_ind = ref - takeoff_window

end_ind = ref

if start_ind < 0:

start_ind = 0

#find takeoff value at the greatest negative deviation from the smoothed signal

ind = np.argmax(MAS[start_ind:end_ind])

if ind == 0:

ind = ind + 1

takeoff_ind = ind + start_ind

AP_takeoff_inds[i] = takeoff_ind

AP_takeoff_vals[i] = data[takeoff_ind]

return AP_takeoff_inds.astype(int), AP_takeoff_vals

def get_rise_time(data, n_peaks, AP_takeoff_inds, AP_peak_inds):

'''Finds rise time of AP phase 0

Parameters

----------

data: input signal (1D numpy array of float32)

n_peaks: number of APs (int)

AP_takeoff_inds: indices of each AP peak (1D numpy array of ints)

AP_peak_inds: indices of each AP peak (1D numpy array of ints)

Returns

----------

AP_rise_times: AP rise times (1D numpy array of ints)'''

MAS = smooth(data) - data

AP_rise_times = np.zeros(n_peaks-1) #allocate space

n_frames = len(data)

for i in range(n_peaks - 1):

#Set search window before takeoff and after peak

start_ind = AP_takeoff_inds[i] - 100

end_ind = AP_peak_inds[i] + 100

if end_ind > n_frames:

end_ind = n_frames - 1

if start_ind < 0:

start_ind = 0

#Find difference between MAS max and min around AP takeoff

MAS_array = MAS[start_ind:end_ind]

AP_rise_times[i] = np.argmin(MAS_array) - np.argmax(MAS_array)

return AP_rise_times.astype(int)

def get_APDmxr(data, n_peaks, AP_peak_inds, AP_peak_vals,

AP_takeoff_inds, AP_takeoff_vals,

AP_peak_deriv1_inds, max_APD = 1000,

mxr_smooth = 100, mxr_window = 100):

'''Uses moving average subtraction to find APDmxr

Parameters

----------

data: input signal (1D numpy array of float32)

n_peaks: number of APs (int)

AP_peak_inds: indices of each AP peak (1D numpy array of ints)

AP_peak_vals: signal value at each AP peak (1D numpy array in float32)

AP_takeoff_inds: indices of each AP peak (1D numpy array of ints)

AP_takeoff_vals: signal value at each AP takeoff (1D numpy array in float32)

AP_peak_deriv1_inds: indices of first derivative peaks for each AP (1D numpy array of ints)

max_APD: sets search window after repolarization to criteria; default = 1000 (int)

mxr_smooth: window size for moving average smoothing; default = 40 (int)

mxr_window: sets search window for APDmxr (int)

Returns

----------

APDmxr_inds: indices of each AP's APDmxr (1D numpy array of ints)'''

APDmxr_inds = np.zeros(n_peaks - 1) #allocate space

MAS = smooth(data, mxr_smooth) - data

#estimate recovery thresholds

crit = (AP_peak_vals - AP_takeoff_vals)*0.25 + AP_takeoff_vals

for i in range(n_peaks - 1):

#Set search window between action potential peak and next action potential takeoff

start_ind = AP_peak_inds[i] + mxr_window

if i == n_peaks - 1:

end_ind = n_frames - 1

else:

end_ind = AP_takeoff_inds[i + 1] - mxr_window

if end_ind < 0:

end_ind = n_frames - 1

if end_ind > AP_peak_deriv1_inds[i] + max_APD:

end_ind = AP_peak_deriv1_inds[i] + max_APD

if end_ind < start_ind:

start_ind = end_ind - mxr_window

if start_ind < 0:

start_ind = 0

#Find first time signal falls below rough recovery threshold

data_arr = data[start_ind:end_ind]

first = get_first(data_arr < crit[i], 0, end_ind - start_ind)

if first == -1:

adj_start_ind = start_ind

else:

adj_start_ind = start_ind + first

#Set repolarization index at the maximum of negative signal deviation

#from smoothed signal after estimated recovery threshold

ind = np.argmax(MAS[adj_start_ind:end_ind])

APDmxr_inds[i] = ind + adj_start_ind

APDmxr = APDmxr_inds - AP_peak_inds[:-1]

return APDmxr.astype(int)

def get_AP_repol_percs(data, n_peaks, AP_peak_inds, AP_peak_vals,

AP_takeoff_inds, AP_takeoff_vals, repol_window = 40):

'''Finds APD30, APD50, APD80

Parameters

----------

data: input signal (1D numpy array of float32)

n_peaks: number of APs (int)

AP_peak_inds: indices of each AP peak (1D numpy array of ints)

AP_peak_vals: signal value at each AP peak (1D numpy array in float32)

AP_takeoff_inds: indices of each AP peak (1D numpy array of ints)

AP_takeoff_vals: signal value at each AP takeoff (1D numpy array in float32)

repol_window: search window size after AP peak (int)

Returns

----------

AP_repol_multi_inds: indices for each AP of repolarization to each target percent

(2D numpy array of ints)'''

repol_percs = [0.3, 0.5, 0.8]

AP_repol_multi_inds = np.zeros((len(repol_percs), n_peaks - 1)) #allocate space

n_frames = len(data)

for repol_idx, repol_perc in enumerate(repol_percs):

crit = (AP_peak_vals - AP_takeoff_vals)*(1-repol_perc) + AP_takeoff_vals

for i in range(n_peaks - 1): #exclude final peak

#set search window

start_ind = AP_peak_inds[i] + repol_window

if i < n_peaks - 1:

end_ind = AP_takeoff_inds[i+1] - repol_window

else:

end_ind = n_frames - 1

#find first time amplitude repolarizes under threshold criteria

if end_ind > start_ind:

data_arr = data[start_ind:end_ind]

first = get_first(data_arr < crit[i], 0, end_ind - start_ind)

#if signal fails to repolarize in window, return minimum signal index

if first == -1:

min_ind = np.argmin(data_arr)

AP_repol_multi_inds[repol_idx, i] = min_ind + start_ind

else:

AP_repol_multi_inds[repol_idx, i] = start_ind + first

else:

AP_repol_multi_inds[repol_idx, i] = 0

APD30 = AP_repol_multi_inds[0,:] - AP_peak_inds[:-1]

APD50 = AP_repol_multi_inds[1,:] - AP_peak_inds[:-1]

APD80 = AP_repol_multi_inds[2,:] - AP_peak_inds[:-1]

return APD30.astype(int), APD50.astype(int), APD80.astype(int)

def analyze_APs(data, default_n_peaks = 20, peak_window_size = 80, takeoff_window = 40,

takeoff_smooth = 100, max_APD = 1000, mxr_smooth = 100,

mxr_window = 100, repol_window = 40):

'''Returns APD30, APD50, APD80, APDtri, APDmxr, and rise time.

Parameters

----------

data: input signal (1D numpy array of float32)

default_n_peaks: estimated number of APs (int)

peak_window_size:

takeoff_window: window size before AP peak for takeoff; default = 40 (int)

takeoff_smooth: width of moving average smoothing function; default = 100 (int)

max_APD: sets search window after repolarization to criteria; default = 1000 (int)

mxr_smooth: window size for moving average smoothing; default = 40 (int)

mxr_window: sets search window for APDmxr (int)

repol_window: search window size after AP peak (int)

Returns

---------

rise_time: phase 0 rise times for each AP (1D numpy array of ints)

APDmxr: time to maximum rate of repolarization for each AP (1D numpy array of ints)

APD30: time to 30% repolarization for each AP (1D numpy array of ints)

APD50: time to 50% repolarization for each AP (1D numpy array of ints)

APD80: time to 80% repolarization for each AP (1D numpy array of ints)

APDtri: APD triangulation (APD80 - APD30) for each AP (1D numpy array of ints)

'''

peak_windows, n_peaks = get_peak_windows(data, default_n_peaks)

AP_peak_deriv1_inds = estimate_peak_idxs(data, peak_windows, n_peaks)

AP_peak_inds, AP_peak_vals = find_AP_peaks(data, n_peaks, AP_peak_deriv1_inds, peak_window_size)

AP_takeoff_inds, AP_takeoff_vals = find_AP_takeoffs(data, n_peaks, AP_peak_deriv1_inds,

takeoff_window, takeoff_smooth)

rise_time = get_rise_time(data, n_peaks, AP_takeoff_inds, AP_peak_inds)

APDmxr = get_APDmxr(data, n_peaks, AP_peak_inds, AP_peak_vals, AP_takeoff_inds, AP_takeoff_vals,

AP_peak_deriv1_inds, max_APD, mxr_smooth, mxr_window)

APD30, APD50, APD80 = get_AP_repol_percs(data, n_peaks, AP_peak_inds, AP_peak_vals,

AP_takeoff_inds, AP_takeoff_vals, repol_window)

APDtri = APD80 - APD30

return rise_time, APDmxr, APD30, APD50, APD80, APDtri

# References

1. O'Hara T, Virag L, Varro A, Rudy Y. Simulation of the undiseased human cardiac ventricular action potential: model formulation and experimental validation. PLoS Comput Biol. 2011;7(5):e1002061. Epub 20110526. doi: 10.1371/journal.pcbi.1002061. PubMed PMID: 21637795; PMCID: PMC3102752.

2. Greenstein JL, Wu R, Po S, Tomaselli GF, Winslow RL. Role of the calcium-independent transient outward current I(to1) in shaping action potential morphology and duration. Circ Res. 2000;87(11):1026-33. PubMed PMID: 11090548.

3. Roth BJ. Mechanisms for electrical stimulation of excitable tissue. Crit Rev Biomed Eng. 1994;22(3-4):253-305. Epub 1994/01/01. PubMed PMID: 8598130.

4. Tomasi C, Manduchi R. Bilateral filtering for gray and color images. Sixth International Conference on Computer Vison (IEEE Cat No98CH36271). 1998:839-46. doi: 10.1109/ICCV.1998.710815.
