# Supplementary material for: Action potential metrics and automated data analysis pipeline for cardiotoxicity testing using optically mapped hiPSC-derived 3D cardiac microtissues
Source: PLoS One. 2023 Feb 6;18(2):e0280406. doi: 10.1371/journal.pone.0280406 (PMC9901774; doi:10.1371/journal.pone.0280406)

COMMENTS FOR AUTHORS:

Reviewer #1: In this manuscript, Arvin H *et al.* developed automated data processing algorithms to assess changes in AP properties for cardiotoxicity testing in 3D engineered cardiac microtissues generated from hiPSC-CMs. This simple and robust automated data analysis pipeline for evaluating key AP metrics provides an excellent *in vitro* cardiotoxicity testing platform for a wide range of environmental and pharmaceutical compounds. Overall, the subject and perspective are novel and valuable for reference. However, to make the review more rigorous and complete, some points must be concerned:

Response: We thank the reviewers for their enthusiasm and positive comments regarding the value of our work to the scientific community. We appreciate their constructive feedback and have addressed the critiques point-by-point below:

Major comments:

1. In this manuscript, 3D organoid is composed by hydrogel aggregation, and whether the established detection system is also suitable for the detection of 3D organoid action potentials in non-hydrogel media or non-conduction media.

Response: We apologize for the confusion and would like to clarify that our 3D cardiac microtissues were constructed under scaffold-free conditions. Specifically, they were created from 95% hiPSC-CMs and 5% human cardiac fibroblasts (by total cell number) that self-assembled and compacted to form beating 3D spheroidal microtissues. The term hydrogel in our manuscript refers to the creation of non-adhesive, agarose template/molds containing 35 cylindrical recesses with rounded bottoms, in which we seed our cells to generate 35 individual microtissues (visualized in "Microtissue Setup" in **Fig 1A**). We have revised the manuscript text (lines 153-157) to clarify this point.

Lines 153-157: To generate scaffold-free 3D cardiac microtissues, purified cardiomyocytes were incorporated with human cardiac fibroblasts (hCFs) at specified ratios (5%, 15%, and 25% of total cell count) before pipetting the cell mixture into the seeding chamber of the agarose hydrogels at a density of 3-5x10^5 cells per hydrogel (~8.5-14x10^4 cells per microwell).

As the reviewer pointed out, it is important to discuss whether the proposed AP analysis approach is applicable/suitable for the detection of APs in 3D cardiac tissues constructed under various scaffold conditions. We would like to acknowledge that our 3D cardiac microtissues likely deposit some extracellular matrix during the 1-week culture period due to fibroblast activation. To address this and further increase rigor, we have added a short paragraph to the discussion section (lines 667-693, repeated below) that details how our AP analysis algorithm is expected to perform in scaffold-based tissues. We also referred to a prior publication from our lab, which employed a similar AP analysis approach to analyze AP properties in cardiac *macro*tissues constructed in a collagen-1, fibrinogen, and thrombin scaffold mixture.

Lines 667-693: Although in this study we optimized the above algorithms to detect changes in AP metrics from optical mapping data of self-assembling hiPSC-derived 3D cardiac microtissues, the

presented algorithms are also applicable when analyzing AP traces from scaffold-based engineered tissues. It is important to note that although our in vitro 3D microtissue model was created under scaffold-free conditions, the addition of hCFs in densely compacting microtissues is likely sufficient to endogenously activate these fibroblasts to deposit extracellular matrix (as reported by others(68)), over the seven-day period of culture. These algorithms are also adaptable for scaffold based engineered tissues of larger scales, with minor modifications in window size. Depending on the pixel resolution of the image, size of the engineered tissue, and tissue conduction velocities, the analysis window size will need to be adjusted accordingly. Since our algorithm is relatively insensitive to increases in window sizes, we do not anticipate this to be an issue. Additionally, depending on the contractile force generated by the engineered tissue, blebbistatin, a potent myosin II inhibitor, may be necessary to minimize motion artifacts. Indeed, we have applied these algorithms to reliably quantify APD metrics from engineered cardiac tissues generated in scaffolds composed of a combination of collagen-1, fibrinogen, and thrombin (69).

2. The layout of the chart can be more optimized and neat. For example, ①-⑤ should be marked in Figure1; A can be directly represented in Figure2 and Fig2.D can be divided into D-G, etc.

Response: We have improved the organization of all our figures, which included adding the markings ①-⑥ in **Fig1B** and dividing **Fig2D** to **Fig 2D-G**. All text referring to these figures have been updated accordingly to reflect these changes. Furthermore, as indicated by Reviewer 2 in minor comments #5-6, we have also increased the clarity/resolution/visibility of our plots by uploading higher resolution vector graphics, increasing the line thickness in our graphs, and recoloring our plots to avoid red/green color combinations.

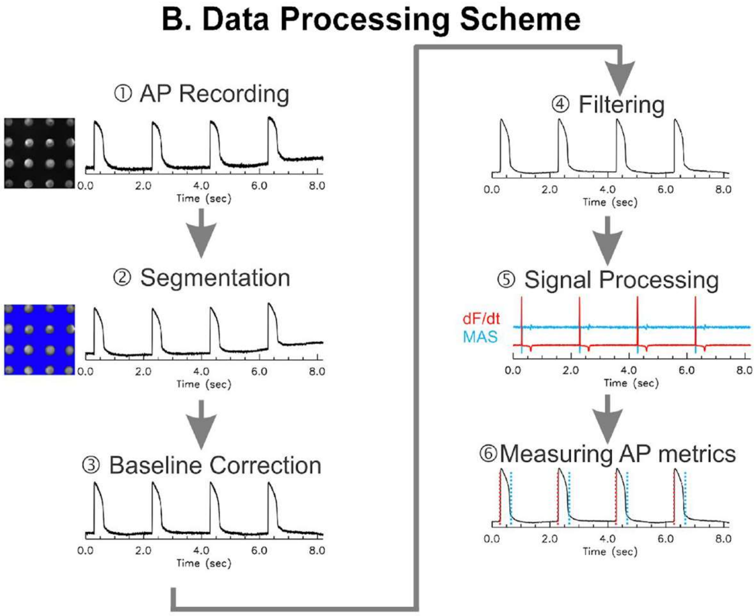

3. In the method part, the writing should be standard and paragraphs should be formatted. For example, "Error! Reference source not found", "A shows" ,etc.

Response: We apologize for this formatting mistake during the PDF conversion process. We have ensured that any errors associated with missing/broken reference links and formatting mistakes have been fixed.

4. Is the ordinate unit (#) in Figure4?

Response: We have added the image intensity label for the ordinate axis (□F/F) in **Fig 4F-G**.

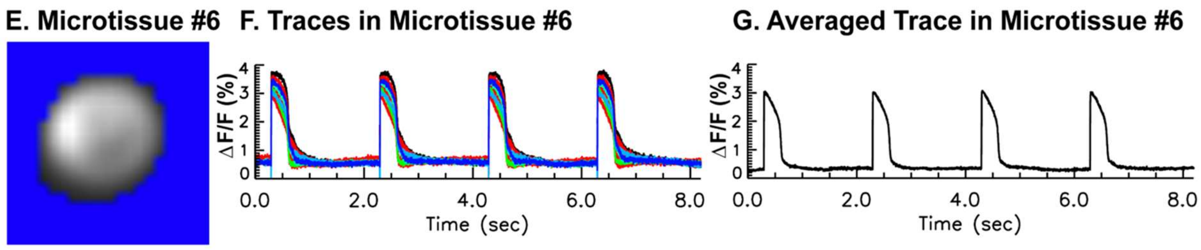

5. The manuscript needs an in-depth review by grammar and spelling with a native speaker.

Response: We have had the manuscript text carefully reviewed by two native English speakers and fixed any grammatical and spelling mistakes.

Reviewer #2: In this study, the authors perform automated optical mapping and analysis to extract action potential metrics and assess cardiotoxicity. Though this is an interesting study and an important endeavor. I have several major comments that must be addressed prior to the acceptance of this manuscript.

Response: Thank you very much for your encouragement and constructive feedback to improve this manuscript. Please see our point-by-point response below:

Major Comments:

1. All code and relevant example data should be provided on GitHub or similar. At present, code snippets are included within a word doc in the supplementary materials of this manuscript. Instead, these functions should be in ".py" files and stored and disseminated on a public GitHub page or similar platform that is designed for sharing code.

Response: We will upload all relevant Python codes, together with the experimentally obtained drug response data set highlighted in **Fig 8**, onto GitHub (link: https://github.com/arvinsoepriatna/AP_Analysis_Routines_Cardiotoxicity_Microtissues) upon acceptance of the manuscript and before the publication deadline. The file size of a single scan from 35 microtissues is 640 Mbytes and we could not upload all the raw data of before and after drugs because of the file size limit. Instead, we were able to create a link to download sample

data. A code/data availability statement has been added to the end of the manuscript and will be updated with the final Github link.

2. In addition to sharing the code as ".py" files, the authors should include full information needed to run both the simulations and analysis code. Specifically, this will require choosing a strategy to share the correct python packages (e.g., requirements.txt file, pyproject.toml file) and installation instructions. And, the authors should provide instructions for running both the simulation code and the analysis software, ideally in the form of a brief tutorial.

Response: We thank the reviewer for this suggestion. The required packages are now listed in the readme file for each routine. Since the data analysis routines are typically linked to the proprietary imaging devices and data acquisition packages, the file structures for import/export may be different, specifically those related to the header structure of data file and data types (char, int, or float points, big or little endian etc). Therefore, we focused on providing each routine as a standalone routine in our distribution package so that users can selectively incorporate each individual routine into their software packages depending on their specific requirements. We provided a Jupyter notebook script as a tutorial so that users can follow the process of each step using the sample data provided.

Lines 749-752: **Data Availability Statement:** All relevant Python codes, together with the experimentally obtained sample data set, presented in this manuscript is made available on GitHub (link: https://github.com/arvinsoepriatna/AP_Analysis_Routines_Cardiotoxicity_Microtissue)

3. One key element missing from the paper is a discussion + quantitative comparison of how these techniques compare to recent open source alternatives shared in the literature. As one (of many) potential examples that should be used for direct comparison, the open source software KairoSight seems to provide similar functionality (e.g., https://github.com/kairosight/kairosight-2.0 and https://www.frontiersin.org/articles/10.3389/fphys.2021.752940/full). The authors should create a table with results from direct comparisons between their proposed methods and alternatives currently used in the literature.

Response: We thank the reviewer for pointing this out and we have added detailed quantitative analysis to compare conventional routines vs. new algorithms proposed in this manuscript. We cited 4 software packages in the Introduction and Discussion sections. In the introduction, we added a rationale why the available packages are difficult to use for cardiotoxicity testing. Most of the available software packages of cardiac electrophysiology aim to map single intact hearts or tissues, making the requirements of image segmentation, filtering, and data analysis are quite different. For example, KairoSight aims to analyze cardiac arrhythmia by mapping electrical propagation and electrophysiological heterogeneity such as APD dispersion from single hearts (apex to base, left and right ventricles, infarct border zone, etc). However, cardiotoxicity testing on hiPSC-derived microtissue platform aims to quickly detect changes in action potential shapes

from multiple microtissues so multiple microtissues are recorded simultaneously as in our platform. Rather than analyzing patterns of action potential propagation or APD heterogeneity within the same heart, AP recordings from a single microtissue were averaged and 8 AP metrics were calculated in this study. Therefore, it is difficult to directly compare individual packages for their performance. Instead, we added quantitative comparisons between typical algorithms used in these software packages vs. new algorithms provided here in this manuscript. Specifically, the following text addressing this comment has been incorporated into the manuscript.

Line 97-107 (Introduction): Several software packages are available for analyzing optical voltage image data of intact hearts or tissues (36-39). These packages are optimized for impulse propagation, dispersion of AP duration (APD), or calcium transient dynamics from the surface of intact hearts to investigate triggered activity, alternans, conduction block, and reentry formation. However, optical mapping of hiPSC cardiac microtissues for cardiotoxicity testing encounters unique hurdles and thus requires different types of data analyses to accurately identify changes in AP shape associated with arrhythmia risks. Unlike whole heart mapping, imaging of microtissues can be accelerated by recording action potentials from multiple samples simultaneously. Higher spatial resolution for voltage imaging of microtissues necessitates intense excitation light that can cause dye bleaching and a loss of fluorescence intensity within the recording time.

Line 626-648 (Discussion): In recent years, open software packages for analyzing voltage data of intact hearts or tissues have been published (36-39). These packages, however, are optimized for impulse propagation, dispersion of APD, or calcium transient dynamics from the surface of intact hearts to investigate triggered activity, alternans, conduction block, and reentry formation. Cardiotoxicity testing in hiPSC microtissue platforms, however, faces a set of different challenges in AP recording. While optical mapping arrhythmia studies in Langendorff perfused hearts often focus on the spatial dynamics of electrical propagation to identify the location of triggered activity and conduction block responsible for reentry formation, cardiotoxicity studies in microtissues focus on detecting small changes in AP parameters to estimate the potential risks of novel drugs in inciting arrhythmias in a dose-dependent manner. As such, a more robust algorithm capable of 1) removing noise while preserving key APs features and 2) sensitively and accurately detecting differences in AP morphologies is necessary for cardiotoxicity microtissue studies. Furthermore, compared to whole heart imaging, data acquisition can benefit from simultaneous recording of APs from multiple microtissues, which requires accurate identification of individual microtissues from a robust automated segmentation approach. Conventional segmentation of optical mapping data employs Otsu's thresholding of fluorescence images, which may work well when imaging intact hearts. However, many in vitro cardiotoxicity platforms employ non-adhesive agarose molds to guide the assembly of 3D microtissues, and these agarose hydrogels often absorb voltage dyes to elevate background fluorescence and reduce image contrast. In this study, image segmentation was improved by FFT and minimization of cross entropy thresholding (Figure 4).

Regarding automated segmentation, our cross entropy algorithm shows consistently lower threshold values that include larger areas of microtissues compared to Otsu's method.

Figure 4 Legend: …. (D) Cross entropy thresholding via a minimum cross entropy algorithm improves the selection of pixels with AP signals (see the red arrow, mean threshold using cross entropy = 30 ± 17 vs. Otsu's method = 72 ± 16, n=30 microtissues). After segmentation, a region-labeling algorithm identified individual microtissues in a solid circular shape compared to Otsu's method in panel C (mean area of microtissue detected using cross entropy = 160 ± 31 pixels vs. Otsu's method = 99 ± 11 pixels, n=30 microtissues). (E) Sample selected microtissue marked with a white box in panel D. (F) Superimposed traces from all the pixels within microtissue #6 in panel E. (G) Averaged trace from all the pixels in panel F.

New plots illustrating the effects of filtering window size on the upstroke velocity, rise time and repolarization measurements are now added to Figure 6 (new bottom panels) and Figure 7 (new panels C and D). These figures show that bilateral filtering (red) preserves the sharpness of AP upstroke better (higher slope value). The rise time (Figure 6C) and repolarization analysis using moving average subtraction (Figure 7C) are a lot less affected by the choice of filtering, which is a significant advantage of our approach compared to Gaussian filtering and derivative approaches.

Figure 6

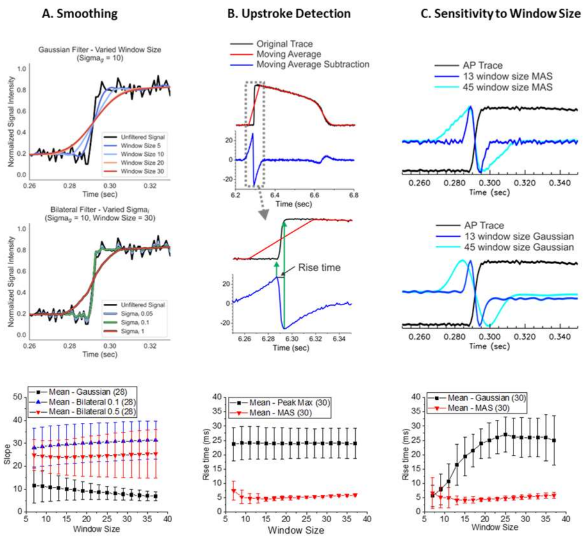

Figure 7

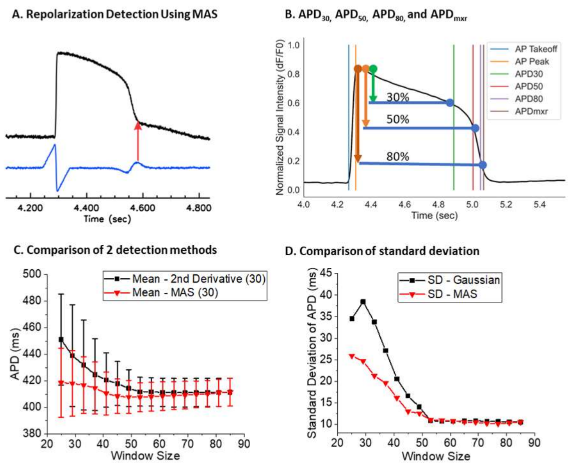

4. On p. 8 in the section "Multi-class logistic regression and principal components analysis" — I encourage the authors to double check a few things and to make sure that they are clear. Specifically: (a) Was the standard scalar fitted using the whole data set or the training data set? (b) Was all hyper parameter tuning done by examining validation data specifically? Based on the description on page 9 line 237 I am concerned that there is overfitting to the small experimental data set. For reference, k-fold cross validation must still have held out test data if the ith fold is being used for hyper parameter and/or model selection. Providing the data sets + scripts in a tutorial format on GitHub or similar will greatly increase my confidence in this part of the work.

Response: We understand the reviewer's concern about overfitting to the small experimental data set. We have added a full data set and Python script for the logistic regression and PCA on GitHub as requested. In response to the reviewer's specific questions: (a) the standard scaler was fit to the training data set, and (b) hyper-parameter tuning was done using the training set with the test set held out. Models were trained on the small experimental data set and the large simulated data set separately. The model trained on the large data set performed more accurately than the model trained on the small data set, which is what we would expect to see if the model trained on the small data set was not overfit. We believe that the model was able to produce highly accurate predictions because the ion channels included in the data set have very distinct roles (ie, AP upstroke was most strongly affected by Na blockade, plateau by Ca channels, and repolarization by hERG channels) and these distinctions were successfully captured by our set of AP metrics. It is possible that logistic regression models would not perform so well at predicting blockades of other ion channels and exchangers that have more complex impacts on cardiac AP. This limitation is now added to the Discussion.

Lines 713-724 (Discussion): The logistic regression model trained on experimental data was able to distinguish Na^+, L-type Ca^{2+}, and hERG channel blocks with high accuracy despite a small-sized data set (*n* = 136, **Figure 8 and Table 4**). The high predictability was also supported by logistic regression models trained on computer modeling data with larger data sets (*n* = 199,983). The high accuracy of the logistic regression model is most likely explained by the sharply distinct roles of the ion channels included in the data sets (i.e., AP upstroke was most strongly affected by Na^+ blockade, plateau by L-type Ca^{2+} channels, and repolarization by hERG channels) and the ability of our set of AP metrics to successfully capture these distinctions. It is possible that logistic regression models would not perform well at predicting blockades of other ion channels and exchangers that have more complex impacts on cardiac AP. Analysis of the computer simulation data sets suggested that I_{to} and I_{Ks} block produce unique changes in AP metrics, although these changes are smaller in magnitude than the changes observed in I_{Na}, I_{Ca}, or I_{Kr} block. However, the PCA fit to simulation data including block of all five channels did not clearly separate I_{to} and I_{Ks} from other channel blocks, largely because effects from I_{to} and I_{Ks} blocks were much smaller in magnitude (**Supplemental Figure S1**) than the effects of I_{Na}, I_{Ca} and I_{Kr} blocks (**Figure 2**). A separate PCA fit to I_{to} and I_{Ks} block simulation data only can separate these two blocks (**Supplemental Figure S1**). Experimentally, I_{Ks} block is difficult to detect with the current protocol in our hiPSC-derived cardiac microtissues as it shows only small changes in AP metrics at a very high concentration of chromanol 293B (**Supplemental Figure S5**). Since sympathetic stimulation increases I_{Ks}, the impact of I_{Ks} on APD is thought to become more significant under sympathetic stimulation (74). Heart rate is also an important factor that can affect $I_{Ks,}$ and the role of I_{Ks} in modulating APD becomes greater at faster pacing cycle length. Further studies will focus on developing a new protocol to detect alteration in I_{Ks} and I_{to} and the associated arrhythmia risks.

Minor Comments:

1. Page 7 line 159: Error! Reference source not found
2. Page 8 line 186: Error! Reference source not found
3. Funding sources listed in the manuscript vs. on the cover page are inconsistent

4. Are all figures and figure panels referenced in the main body of the paper? It is hard to keep track especially with the error in referencing.
5. Many of the figures are pixelated — this should be addressed.
6. The figure color scheme should be adjusted to be friendly to readers with red/green colorblindness.

Response: We have fixed all the errors related to broken reference sources and ensured that all figure panels were referenced in the main body of the paper in the appropriate order. We made sure that funding sources listed in the manuscript and the cover page are consistent. High-quality vector graphics have been uploaded to improve image resolution, and figure color schemes have been edited with different shapes (i.e. squares, triangles, and circles) and line types to assist readers with red/green colorblindness. Modified Figure 2D-G and Figure 8E are shown below.

Figure 2 D-G

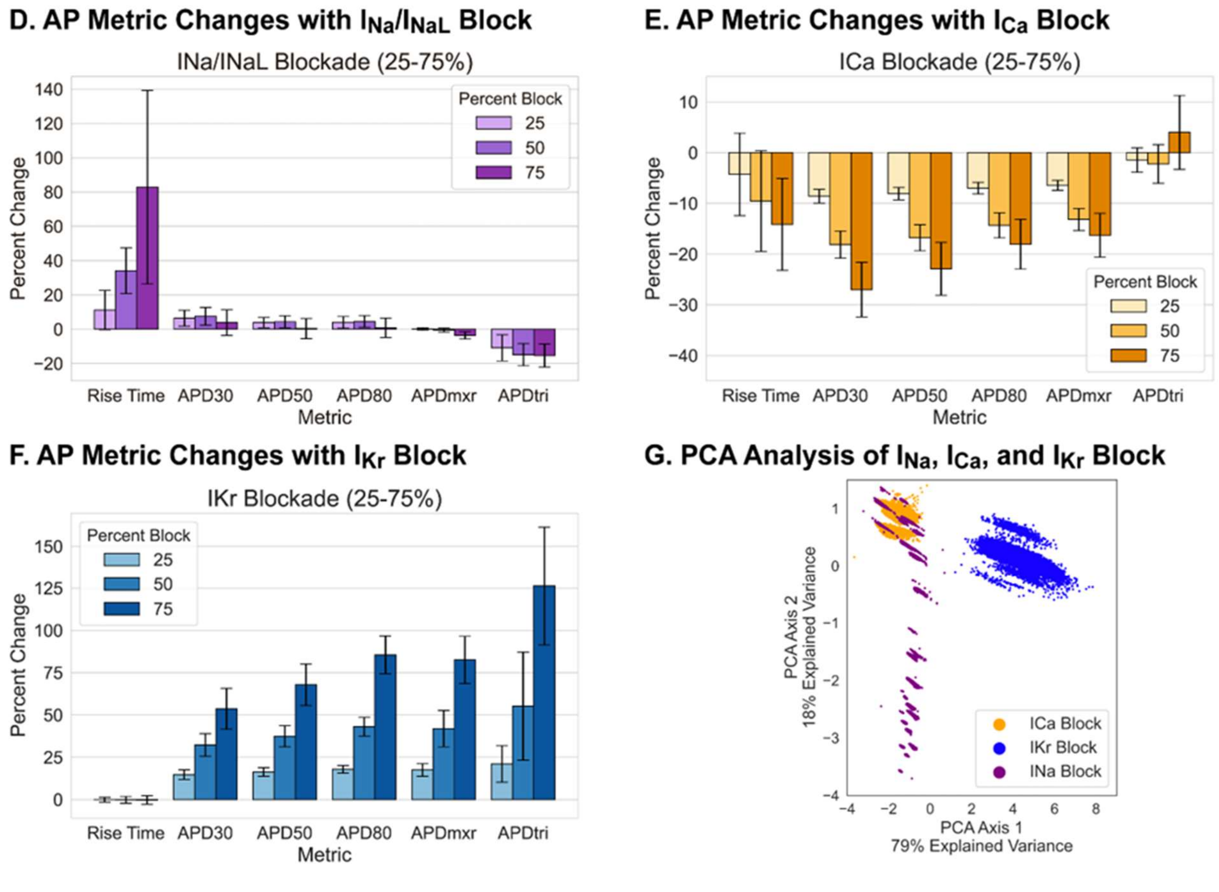

Figure 8E

E. PCA of AP Metric Changes by TTX, Flecainide, Nifedipine, and E4031

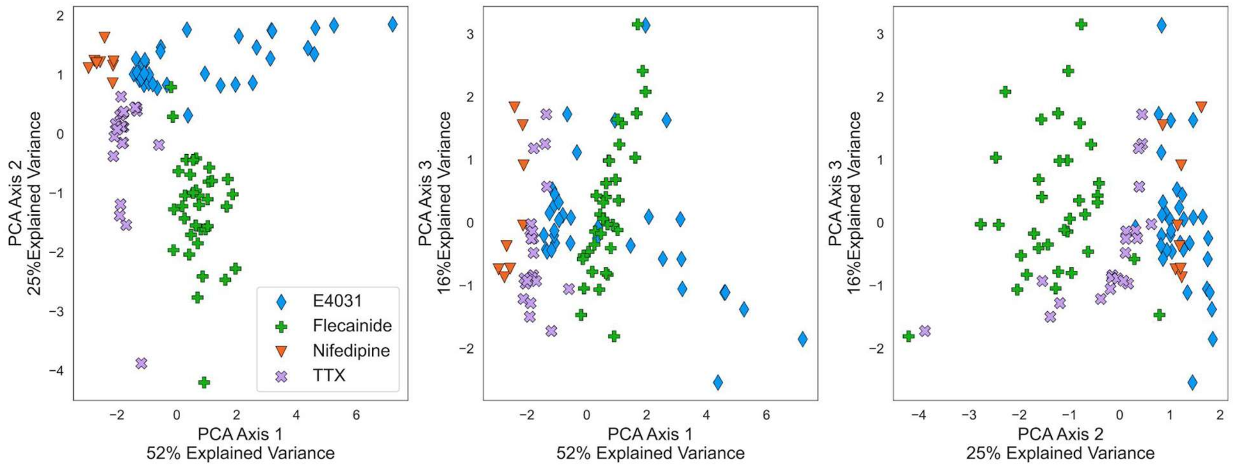

Supplement: Attachment — Submitted filename: PLoS One Reponse to Reviewer - Cardiac AP Algorithm Paper.pdf [file pone.0280406.s003.pdf]
